# Discrimination of superficial lymph nodes using ultrasonography and tissue metabolomics coupled with machine learning

**DOI:** 10.3389/fonc.2025.1510018

**Published:** 2025-01-28

**Authors:** Lu Li, Xinyue Wang, Hongyan Deng, Wenjuan Lu, Yasu Zhou, Xinhua Ye, Yong Li, Jie Wang

**Affiliations:** ^1^ Department of Ultrasound, The First Affiliated Hospital, Nanjing Medical University, Nanjing, China; ^2^ Institute of Food Safety and Nutrition, Jiangsu Academy of Agricultural Sciences, Nanjing, China; ^3^ Department of Radiology, The First Affiliated Hospital, Nanjing Medical University, Nanjing, China

**Keywords:** lymph nodes, lymphoma, metastasis, ultrasonography, metabolomics

## Abstract

**Introduction:**

Diagnosing the types of malignant lymphoma could help determine the most suitable treatment, anticipate the probability of recurrence and guide long-term monitoring and follow-up care.

**Methods:**

We evaluated the differences in benign, lymphoma and metastasis superficial lymph nodes using ultrasonography and tissue metabolomics.

**Results:**

Our findings indicated that three ultrasonographic features, blood supply pattern, cortical echo, and cortex elasticity, hold potential in differentiating malignant lymph nodes from benign ones, and the shape and corticomedullary boundary emerged as significant indicators for distinguishing between metastatic and lymphoma groups. Metabolomics revealed the difference in metabolic profiles among lymph nodes. We observed significant increases in many amino acids, organic acids, lipids, and nucleosides in both lymphoma and metastasis groups, compared to the benign group. Specifically, the lymphoma group exhibited higher levels of nucleotides (inosine monophosphate and adenosine diphosphate) as well as glutamic acid, and the metastasis group was characterized by higher levels of carbohydrates, acylcarnitines, glycerophospholipids, and uric acid. Linear discriminant analysis coupled with these metabolites could be used for differentiating lymph nodes, achieving recognition rates ranging from 87.4% to 89.3%, outperforming ultrasonography (63.1% to 75.4%).

**Discussion:**

Our findings could contribute to a better understanding of malignant lymph node development and provide novel targets for therapeutic interventions.

## 1 Introduction

Lymphadenopathy can result from a myriad of causes, encompassing infections, autoimmune diseases, neoplasms, hematological disorders, and more (1). Malignant lymph nodes include metastases and lymphoma (2). Diagnosing the specific type of malignant lymphoma could help determine the most appropriate treatment, anticipate the probability of recurrence and guide long-term monitoring and follow-up care (3). Various imaging techniques have been employed for the diagnosis of malignant lymph nodes, including computed tomography (CT), magnetic resonance imaging (MRI), positron emission tomography-computed tomography (PET-CT), and ultrasonography (US). Among these imaging techniques, ultrasonography stands out due to cost-effectiveness, operational ease, and ability to provide high-resolution information (4). Ultrasonography utilizes morphological criteria to discern benign from malignant lymph nodes (5–7). Malignancies often manifest as lymph nodes that exhibit enlarged size, heterogeneous internal structure, an absence of echogenic hilum, compromised capsule integrity, and a peripheral vascular pattern (6). However, certain malignant lymph nodes with typical morphology could be easily misdiagnosed in ultrasonographic examinations (8). Moreover, in cases of lymph node enlargement, it can be challenging to distinguish reactive hyperplasia from metastatic occurrences (9). Therefore, there is an urgent need to deeply understand the difference in the ultrasound characteristics of malignant lymph nodes with various pathological categories.

Metabolomics encompasses the high-throughput identification and quantification of small molecule metabolites, which are the end products of metabolism, in biological samples (10). Metabolomics has been utilized in human medicine and clinical research. In contrast to traditional imaging techniques, metabolomics is emerging as a powerful tool for comprehending disease mechanisms, discovering biomarkers, and advancing personalized medicine (11, 12). For instance, Schmidt et al. (13) demonstrated that acylcarnitine C18:1, citrulline, trans-4-hydroxyproline and three glycerophospholipids in blood may have associations with prostate cancer (13). Wang et al. (14) found that patients with Parkinson’s disease exhibited elevated levels of 3-methoxytyramine, N-acetyl-L-tyrosine, orotic acid, uric acid, vanillic acid, and xanthine in their urine (14). Shao et al. (29) found the higher levels of metabolic intermediates and the enrichment of genes involved in the tricarboxylic acid (TCA) cycle in prostate cancer tissues (15). However, reports remain limited concerning alterations in metabolic profiling of superficial lymph nodes across different pathologies.

In this study, benign, lymphoma, and metastatic lymph nodes were analyzed using ultrasonography and LC-QTOF/MS-based tissue metabolomics. Statistical analysis was applied to identify variations in ultrasound features and metabolites among the lymph node groups. The main objective of this study was I) to investigate the difference in metabolic and ultrasound characteristics among superficial lymph nodes with different pathological types and II) to compare the diagnostic efficiency of the two methods for discriminating superficial lymph nodes. Our findings could contribute to a better understanding of malignant lymph node development and offer potential biomarkers for diagnosing malignant lymph nodes.

## 2 Materials and methods

### 2.1 Patients and sampling

A total of 78 patients with superficial lymph node enlargement were enrolled at the first affiliated hospital of Nanjing medical university from February 2021 to June 2022. All patients underwent ultrasonography of lymph nodes. If the cause of lymphadenopathy could not be diagnosed, the patient would undergo an ultrasound-guided needle biopsy. The criteria for inclusion were patients assessed by the sonographer and clinician requiring ultrasound-guided needle biopsy. The exclusion criteria included: I) contraindications for a core needle biopsy, such as uncontrolled acute infection and apparent tendency to develop activity; II) incomplete pathological results (Routine pathological diagnosis was incomplete and immunohistochemical results were lacking); and III) borderline diseases, such as Castleman disease.

Lymph node tissue was biopsied with a 16G needle under ultrasound guidance and four specimens of tissue were obtained. Three specimens were used for pathological examination, and one was stored in a -80°C for metabolomic analysis. According to inclusion and exclusion criteria, 69 patients were up to the standard, and these samples were subjected to metabolomic analysis. According to immunohistochemical pathology, the lymph nodes were divided into benign (N=20), lymphoma (N=29), and metastasis (N=20) groups.

The ethics committee of the first affiliated hospital of Nanjing medical university has approved this study. The ethics committee of the First Affiliated Hospital of Nanjing Medical University has approved this study. Prior to participation, all patients signed an informed consent form, and investigators securely protected and analyzed all data.

### 2.2 Ultrasound features

Ultrasound examination and ultrasound-guided core needle biopsy were respectively performed by two doctors with ten years of experience in ultrasound diagnosis and interventional. Conventional ultrasound images were obtained by Philips Epiq 5 (L12-5, 5–12 MHz) and Super Sonic Imagine Aixplorer-1 (SL15-4, 4–15 MHz), and ultrasound-guided core needle biopsy using GE Logiq E9 (9L, 3–10 MHz). Ultrasound characteristics for each lymph nodes were collected: long diameter, short diameter, shape, vascular pattern, hilum, cortical thickness, cortical echo, cortex elasticity, corticomedullary boundary, ratio of short to long, calcification, elasticity, Adler grade of blood flow were recorded. The assignment of variables was shown in **Table 1**. The elastography was assessed using ChoiJJ’s 4-point method: 1 point for all lymph nodes being green or bluish, 2 points for scattered blue areas comprising less than 45% proportion, 3 points for blue areas larger than 45%, and 4 points for the blue area occupying the entire lymph node with or without a green border. Furthermore, 1~2, 3 and 4 points is soft, medium and hard, respectively.

**Table 1:** Information on the variable assignment.

Factors	Assignment
Gender	0 for male; and 1 for female
Shape	0 for regular; and 1 for irregular
Hilum	0 for preserved; 1 for partially preserved; and 2 for completely obliterated
Cortical thickness	0 for normal; and 1 for thickened
Cortical echo	0 for homogeneous isoecho; 1 for reticular; and 2 inhomogeneous hyperecho
Corticomedullary boundary	0 for clear; and 1 for unclear
Ratio of short to long	0 for <0.5; and 1 for ≥ 0.5
Calcification	0 for none; 1 for microcalcification; and 3 for macrocalcification
Lymph node fusion	0 for none; and 1 for confluent
Vascular pattern	0 for hilar vascularity; 1 for mixed vascularity; and 2 peripheral vascularity
Adler grade of blood	0 for Grade 0; 1 for Grade I; 2 for Grade II; and 3 for Grade III
Elastography	0 for soft; 1 for medium; and 2 for hard

### 2.3 Metabolomic analysis

Each lymph node sample was extracted with 2-mL 80% methanol solution, rotated for 10 min and centrifuged at 8000×g for 20 min. The supernatant was filtered through a 0.22-µm membrane filter and analyzed using LC-QTOF/MS (TripleTOF 5600+, AB SCIEX). The MS instrument was equipped with an electrospray ionization source and each sample was detected by the mass spectrometry under positive and negative ionization modes. The full-scan mode (50-1000 m/z) based on information-dependent acquisition mode was applied. Mobile phase A in the positive and negative ionization modes were 0.1% formic acid/water and 5mM ammonium acetate, respectively and the mobile phase B in both two modes was acetonitrile. The gradient elution process of mobile phase B was at 1% (v/v) for 0-3 min, 1%-99% (v/v) for 3-21 min, 99% (v/v) for 21- 28 min, and 1% (v/v) for 28-34 min. The ion spray voltage and collision energy were 5500 V and 35 V, respectively, in the positive ionization mode and were −4500 V and -35 V, respectively, in the negative ionization mode. The nebulizer, curtain gas, and heater flow pressures were set to 50, 25, and 50 psi, respectively. Besides, a quality control (QC) sample was prepared by mixing each lymph node sample in equal volume (10 μL) was detected every 6 samples under the above condition.

The open source software MS-DIAL was used to analyze mass spectrometry data. The MS peaks were qualitatively analyzed by comparing the similarity of the first- and second-order mass spectrometry, and retention time in publicly available databases (including MassBank, GNPS, MetaboBASE, and LipidBlast) and some of them were further confirmed with standard chemicals (**Supplementary Table S1**). Principal component analysis (PCA) was used to analyze the differences of metabolic profiling among three groups. The PLS-DA model with VIP greater than 1 and t-test with P-value less than 0.05 were used to screen the metabolites with significant changes. The identified metabolites with significant change was applied for metabolic pathway analysis using by a web-based tool MetaboAnalysts based on the database Kyoto Encyclopedia of Genes and Genomes. *Pearson*’s correlation analysis analyzed the correlation of identified metabolites with ultrasound features and patient’s age and gender, with a significance level set at *P* < 0.05.

### 2.4 Discriminant model

Linear discriminant analysis was applied in conjunction with ultrasonography or tissue metabolomics to differentiate benign, lymphoma, and metastasis lymph nodes. For metabolomics, a random forest analysis was conducted to measure variable importance of metabolites for distinguishing the superficial lymph nodes. Within each group, 90% and 10% of samples were randomly allocated to training and prediction sets, respectively, utilizing the Monte Carlo cross-validation method. For mitigating the error from one single calculation, Monte Carlo simulation was run 100 times and the average diagnostic rate of each groups was calculated.

## 3 Results

### 3.1 Diagnostic efficacy of ultrasonographic features

**Figure 1:**
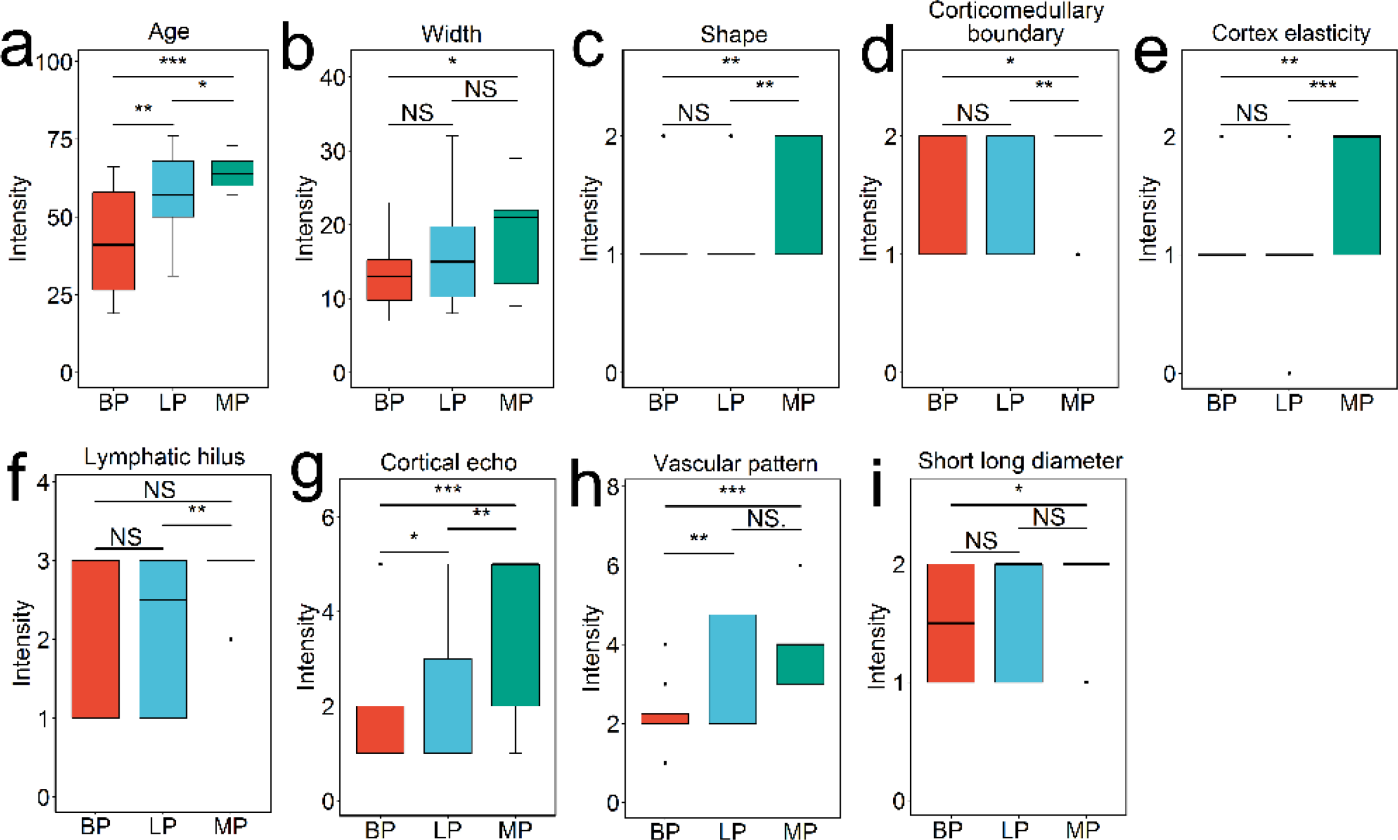
Patient and ultrasonography indices with significant difference among benign, lymphoma, and metastasis groups, analyzed using Student’s *t*-test. The features include age **(A)**, width **(B)**, shape **(C)**, corticomedullary boundary **(D)**, cortex elasticity **(E)**, hilus **(F)**, cortical echo **(G)**, vascular pattern **(H)**, and short long diameter **(I)**. NS indicates no significance (*P* > 0.05); * indicates *P* < 0.05; ** indicates *P* < 0.01; *** indicates *P* < 0.001.

For each superficial lymph nodes, the information of ultrasound sonographic characteristics and patients’ age and gender were collected. The result of t-test analysis revealed that there were significant differences in these indices between three groups. Three indices, age, vascular pattern and cortical echo, were significantly varied between lymphoma and benign groups (*P* < 0.05) (**Figure 1**). Nine indices, age, width, shape, vascular pattern, hilum, cortical echo, cortex elasticity, corticomedullary boundary and short long diameter were significantly changed between metastasis and benign groups (*P* < 0.05) (**Figure 1**). There were significant differences in six indices between lymphoma and metastasis groups, including age, cortex elasticity, shape, cortical echo, hilum and corticomedullary boundary (*P* < 0.05).

### 3.2 Metabolic profiling analysis

**Figure 2:**
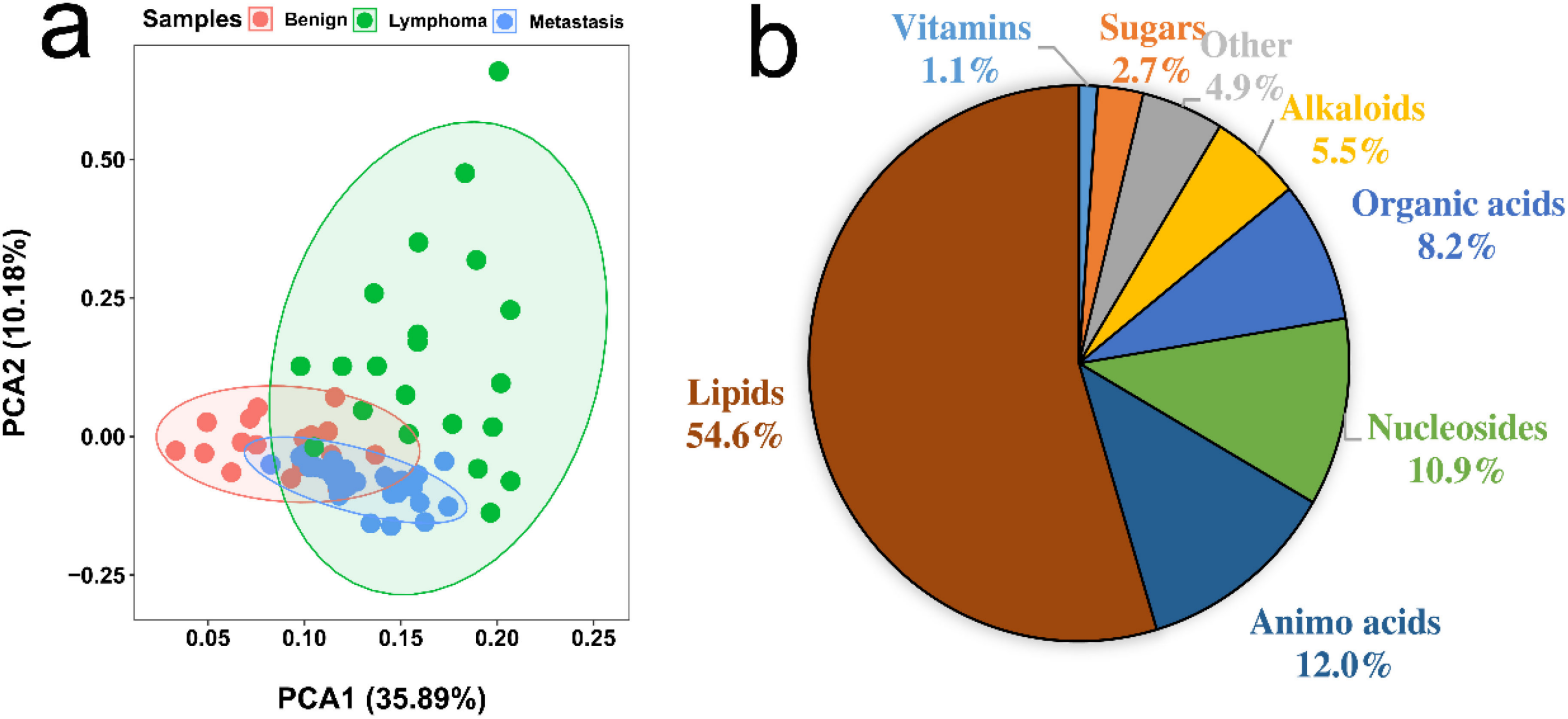
**(A)** PCA analysis of metabolic profiling of benign, lymphoma, and metastasis lymph nodes. **(B)** Proportion of identified metabolites that showed significant changes, determined by Student’s *t*-test (*P* < 0.05) and PLS-DA (VIP > 1).

**Figure 3:**
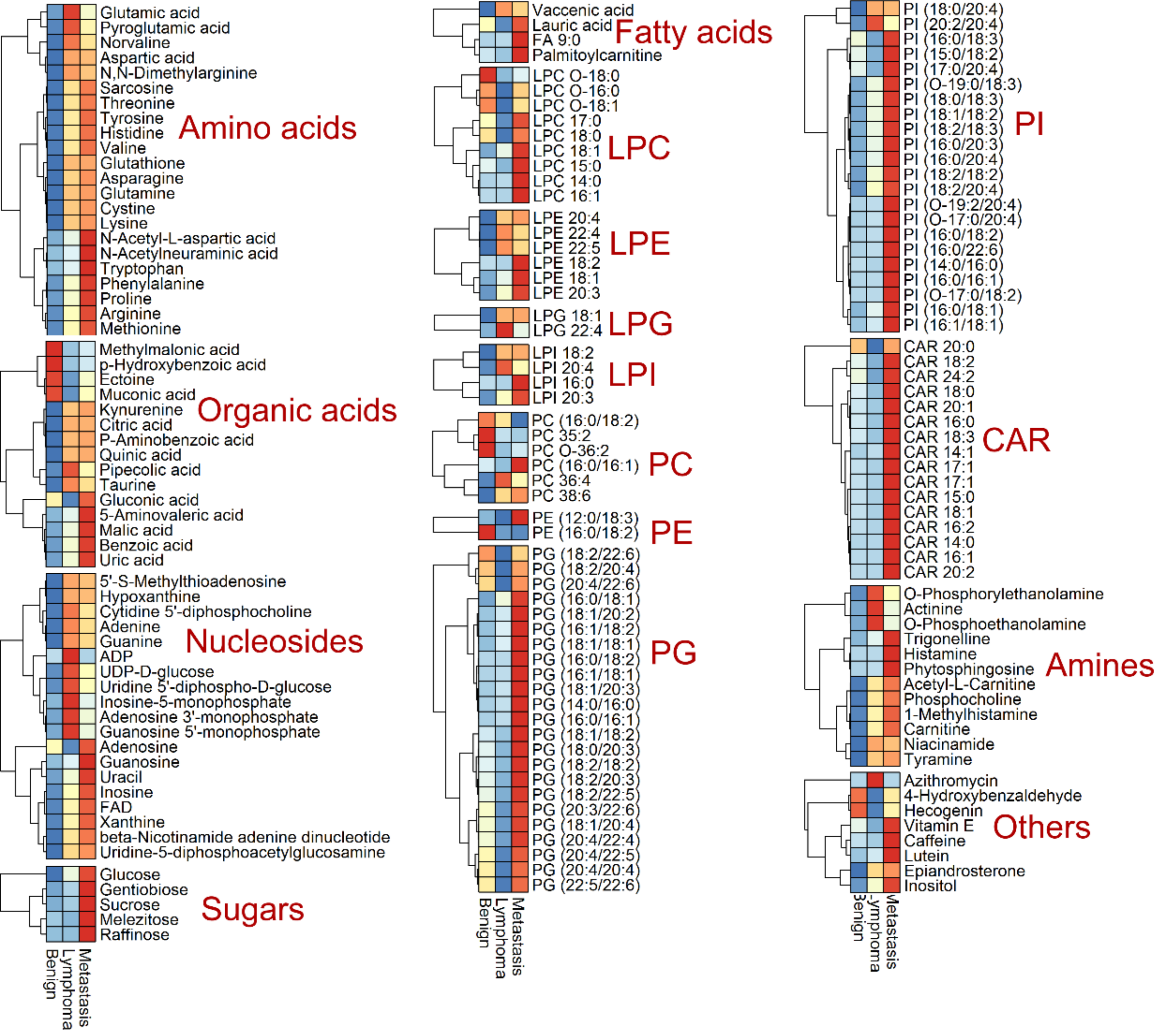
Heatmap analysis of identified metabolites with significant change, including amino acids, organic acids, nucleosides, sugars, lipids (fatty acids, lysophosphatidylcholine (LPC) lysophosphatidylethanolamine (LPE), lysophosphatidylinositol (LPI), lysophosphatidylglycerols (LPG), phosphatidylcholine (PC), phosphatidylethanolamine (PE), phosphatidylinositol (PI) and phosphatidylglycerol (PG) and acylcarnitine (CAR)), amines and others.

We conducted metabolic profiling of lymph node samples using LC-QTOF/MS analysis. The total ion chromatograms of a lymph node sample are shown in **Supplementary Figures S1**, **S2**. MS-DIAL software was utilized to analyze the data, resulting in the detection of 6754 peaks from the positive mode and 3474 peaks from the negative mode. Principal component analysis (PCA) was performed, showing that the first two principal components explained 46.07% of the total variance (**Figure 2A**). The PCA plot based on these components clearly displayed differences in the metabolic profiles among the three groups of lymph nodes (**Figure 2A**). Using PLS-DA with a VIP value > 1 and t-test with a *P*-value < 0.05, we identified a total of 174 metabolites with significant changes. These metabolites primarily belonged to categories such as amino acids, organic acids, nucleosides, lipids, sugars, and amines (**Figure 2B**; **Supplementary Table S1**). Notably, lipids were found to be the most abundant metabolites, including fatty acids, lysophospholipids (lysophosphatidylcholine (LPC) lysophosphatidylethanolamine (LPE), lysophosphatidylinositol (LPI), and lysophosphatidylglycerols (LPG)), glycerophospholipids (phosphatidylcholine (PC), phosphatidylethanolamine (PE), phosphatidylinositol (PI) and phosphatidylglycerol (PG)) and acylcarnitine (CAR). The heatmap plot of these metabolites with significant change was shown in **Figure 3**.

### 3.3 Differential metabolite analysis

**Figure 4:**
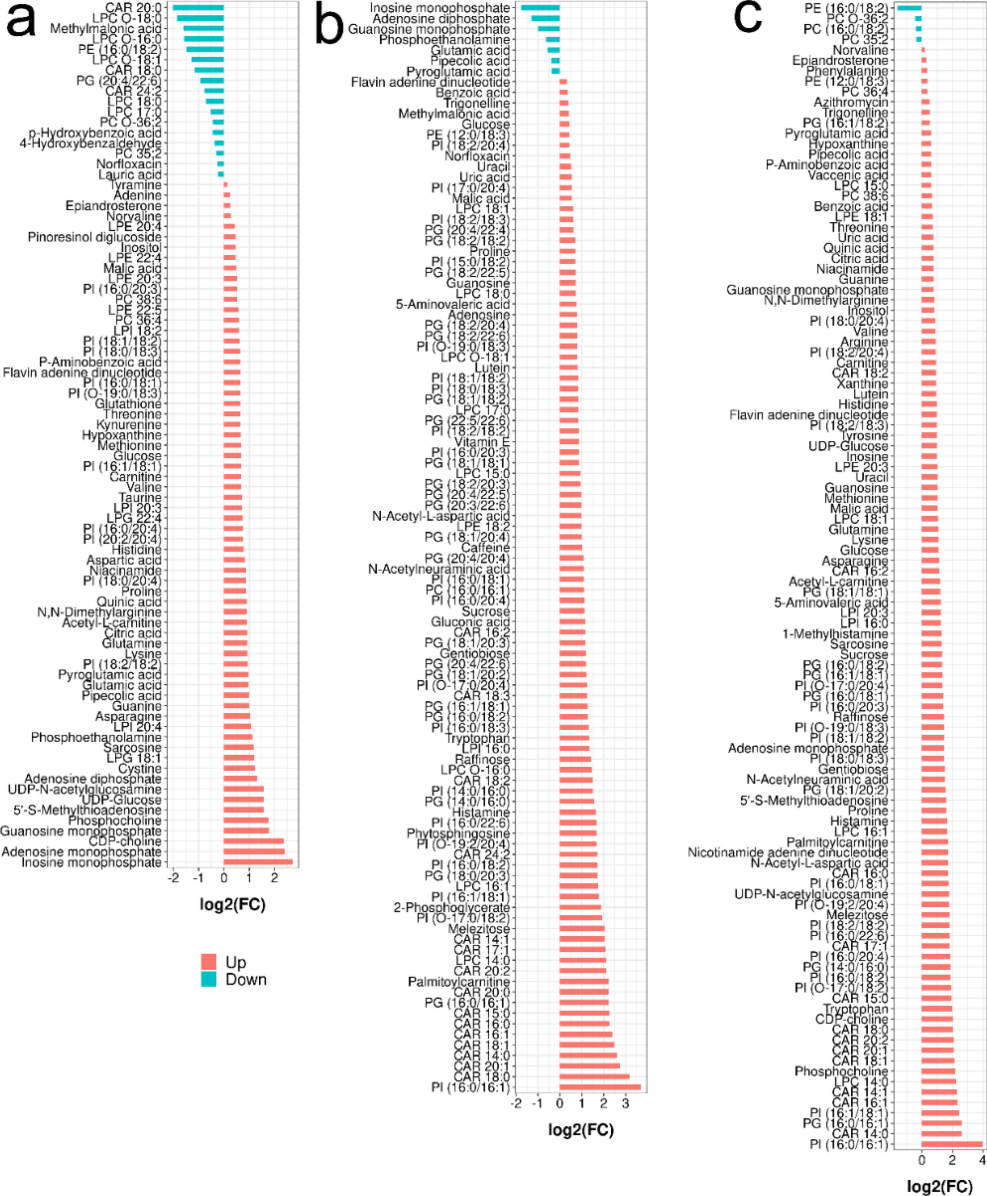
Barplot analysis of the identified metabolites with significant change in different comparison (Student’s *t*-test with *P* < 0.05, PLS-DA with VIP > 1): **(A)** for lymphoma versus benign, **(B)** for metastasis versus lymphoma and **(C)** for metastasis versus benign.

In the lymphoma versus benign group, 66 metabolites were significantly upregulated, mainly amino acids, nucleosides and amines (**Figure 4A**). Phosphocholine, guanosine monophosphate, cytidine diphosphocholine, adenosine monophosphate and inosine monophosphate increased the most, with fold changes greater than 3.0. A total of 17 metabolites were significantly decreased, and CAR 20:0, LPC O-18:0, methylmalonic acid, LPC O-16:0, PE (16:0/18:2), LPC O-18:1, and CAR 18:0 had the largest decrease at 0.246~0.448 times.

When comparing metastasis and lymphoma groups, 96 metabolites were significantly decreased, mainly including lipids and sugars (**Figure 4B**). Palmitoylcarnitine, CAR 16:0, CAR 20:0, CAR 15:0, CAR 16:1, CAR 18:1, CAR 14:0, CAR 20:1, CAR 18:0, PI (16:0/16:1) increased the most, by more than 5.0 times. Nine metabolites were significantly downregulated, mainly including inosine monophosphate, actinine, glutamic acid, phosphoethanolamine and pipecolic acid (ranging from 0.208- and 0.734-fold).

In metastasis versus benign groups, 102 metabolites were significantly upregulated, mainly including amino acids, nucleosides, sugars and lipids (**Figure 4C**). PI (16:0/16:1) had the largest increase at 17.35-fold, followed by CAR 14:0, PI (16:1/18:1), PG (16:0/16:1), CAR 16:1, CAR 14:1, LPC 14:0, phosphocholine, CAR 18:1, CAR 20:1, CAR 18:0, CAR 20:2 and tryptophan; only 4 metabolites were significantly downregulated.

### 3.4 Metabolic pathways analysis

**Figure 5:**
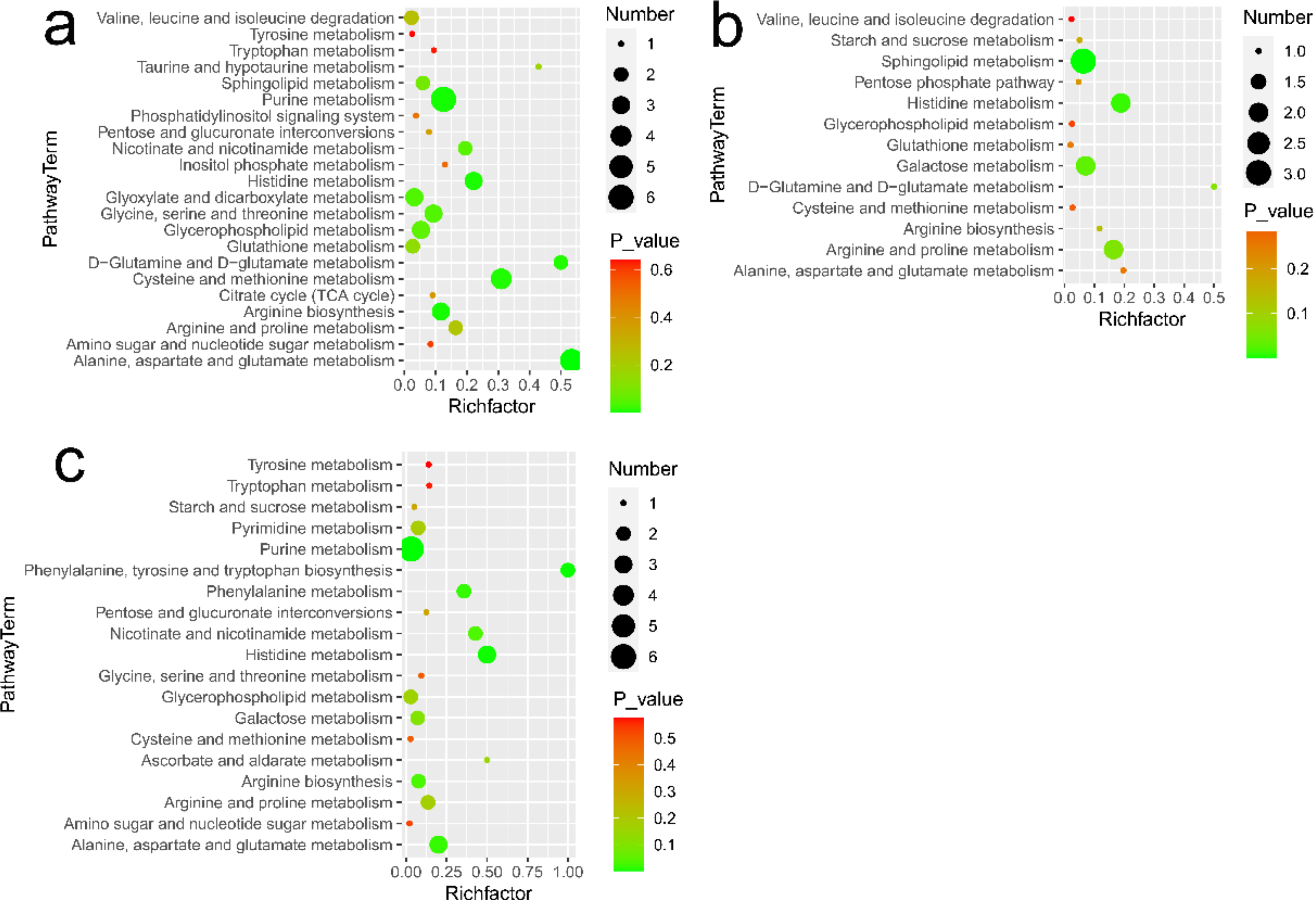
Metabolic pathways analysis in different comparisons: **(A)** for lymphoma versus benign groups, **(B)** for metastasis versus lymphoma groups, and **(C)** for metastasis versus benign groups. Number represents the number of metabolites matched from the data and *P*-value represents *P* value calculated from pathway analysis.

Based on MetaboAnalyst and metabolites with significant changes, the enriched metabolic pathways in lymphoma versus benign, metastasis versus lymphoma, and metastasis versus benign groups are showed in **Figures 5A–C**, respectively. The metabolic pathways, including alanine, aspartate and glutamate metabolism, arginine and proline metabolism, histidine metabolism, glycerophospholipid metabolism, were commonly enriched in all comparisons. Besides, D-glutamine and D-glutamate metabolism, valine, leucine and isoleucine degradation, citrate cycle, phosphatidylinositol signaling system, glutathione metabolism, and glyoxylate and dicarboxylate metabolism were additionally enriched in benign versus lymphoma groups. Phenylalanine, tyrosine and tryptophan biosynthesis, ascorbate and aldarate metabolism and pyrimidine metabolism were additionally enriched in benign versus metastasis groups. Starch and sucrose metabolism and galactose metabolism were additionally enriched in lymphoma versus metastasis groups.

### 3.5 Linear discriminant analysis

Linear discriminant analysis coupled with ultrasonography indices or metabolites with significant difference was used to differentiate superficial lymph nodes. Two discriminant functions were developed, where the first one was used to differentiate benign groups from all samples, while the second one focused on distinguishing between lymphoma and metastasis groups. For ultrasonography, 77.1% of benign nodes, 66.7% of lymphoma nodes and 64.5% of metastasis nodes in the training set were correctly classified (**Table 2**). Lymphoma and metastasis nodes were commonly misclassified as one another, with 23.7% of lymphoma nodes incorrectly classified as metastasis and 31.5% of metastasis nodes misclassified as lymphoma. Overall, 68.6% of all nodes were accurately classified using linear discriminant analysis and ultrasonography.

**Table 2:** Diagnostic rates of LDA with ultrasonography and metabolomics.

Data	Class		Ultrasonography			Metabolomics	
		Benign	Lymphoma	Metastasis	Benign	Lymphoma	Metastasis
Training set	Benign	77.1	10.3	12.6	90.1	4.6	5.3
	Lymphoma	6.9	66.7	26.4	3.2	88.5	8.3
	Metastasis	4.5	31	64.5	2.7	5.9	91.4
Testing set	Benign	75.1	12.3	12.6	88.5	3.7	7.8
	Lymphoma	8.4	67.9	23.7	5.6	87.4	7.0
	Metastasis	5.4	31.5	63.1	4.1	6.6	89.3

**Figure 6:**
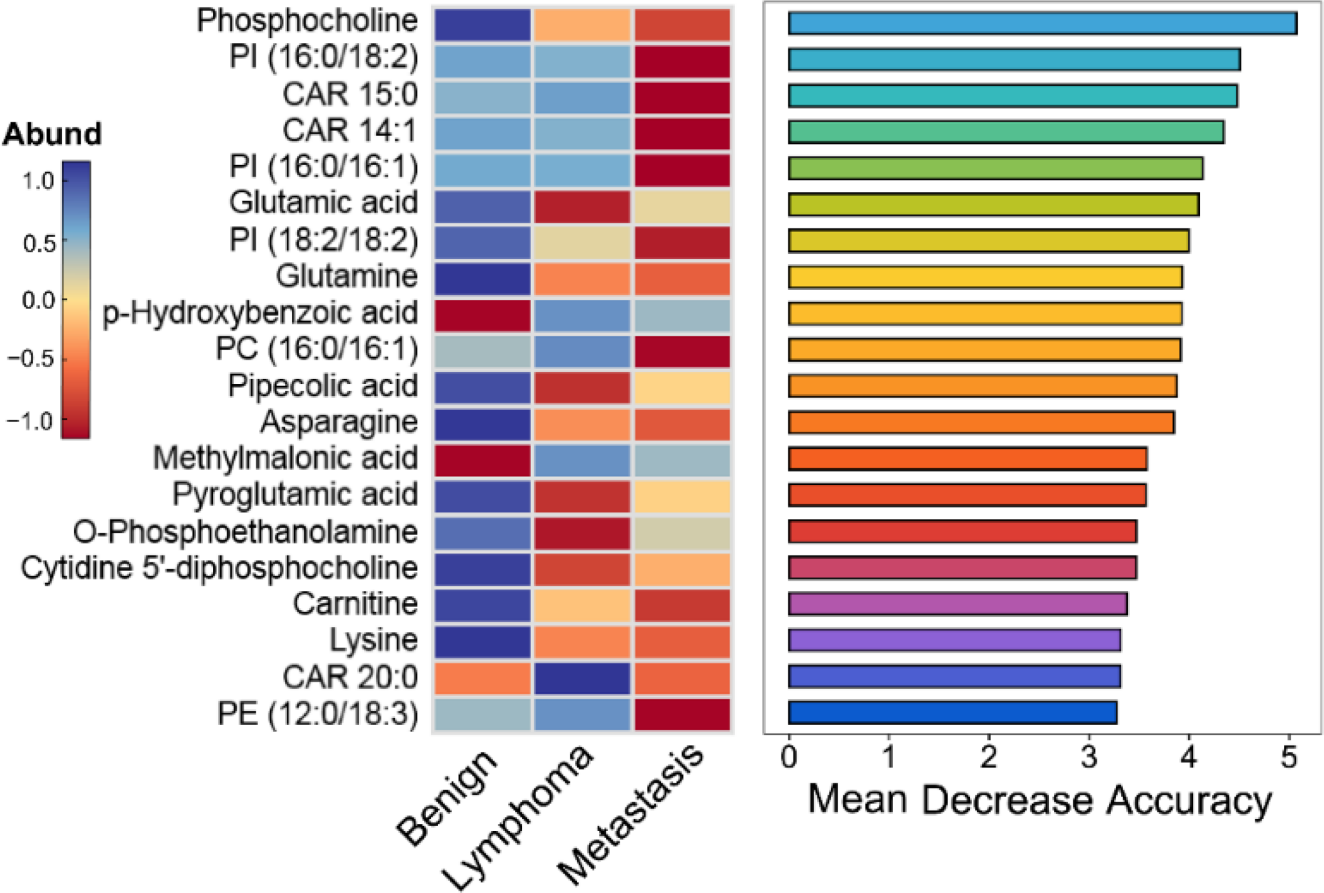
Top 20 important metabolites identified by the Mean Decrease Accuracy of random forest analysis for distinguishing different superficial lymph nodes.

There existed many metabolites with significant difference among three groups. However, too many variables could negatively impact the performance of diagnostic models. Additionally, building model based on a great number of metabolites may hinder practical applications. Therefore, it is essential to select a subset of important metabolites for modeling. Random forest is a commonly used method for variable selection in metabolomics. The top 20 important metabolites for distinguishing superficial lymph nodes based on Mean Decrease Accuracy in Random forest (**Figure 6**). These include 5 amino acids (glutamic acid, glutamine, asparagine, lysine and pyroglutamic acid), 8 lipids [CAR 14:1, CAR 15:0, CAR 20:0, PC (16:0/16:1), PE (12:0/18:3), PI (16:0/16:1), PI (16:0/18:2) and PI (18:2/18:2)], 3 organic acids (methylmalonic acid, p-hydroxybenzoic acid and pipecolic acid), and 3 alkaloids (O-phosphoethanolamine, carnitine and phosphocholine). Linear discriminant analysis coupled with these 20 metabolites showed that 90.1% of benign nodes, 88.5% of lymphoma nodes and 91.4% of metastasis nodes in the training set were correctly classified. In the testing set, the diagnostic efficiency of metabolomics was acceptable, where 88.5% of benign nodes, 87.4% of lymphoma nodes and 89.3% of metastasis nodes in the training set were correctly classified. The diagnostic accuracy of superficial lymph nodes in the training set were comparable to that in testing set, suggesting that the models developed were robust and did not exhibit overfitting. Overall, 88.3% of all nodes were correctly classified using metabolomics, which was much higher than that of ultrasonography (68.6%).

### 3.6 Correlation analysis between ultrasound features and metabolites

**Figure 7:**
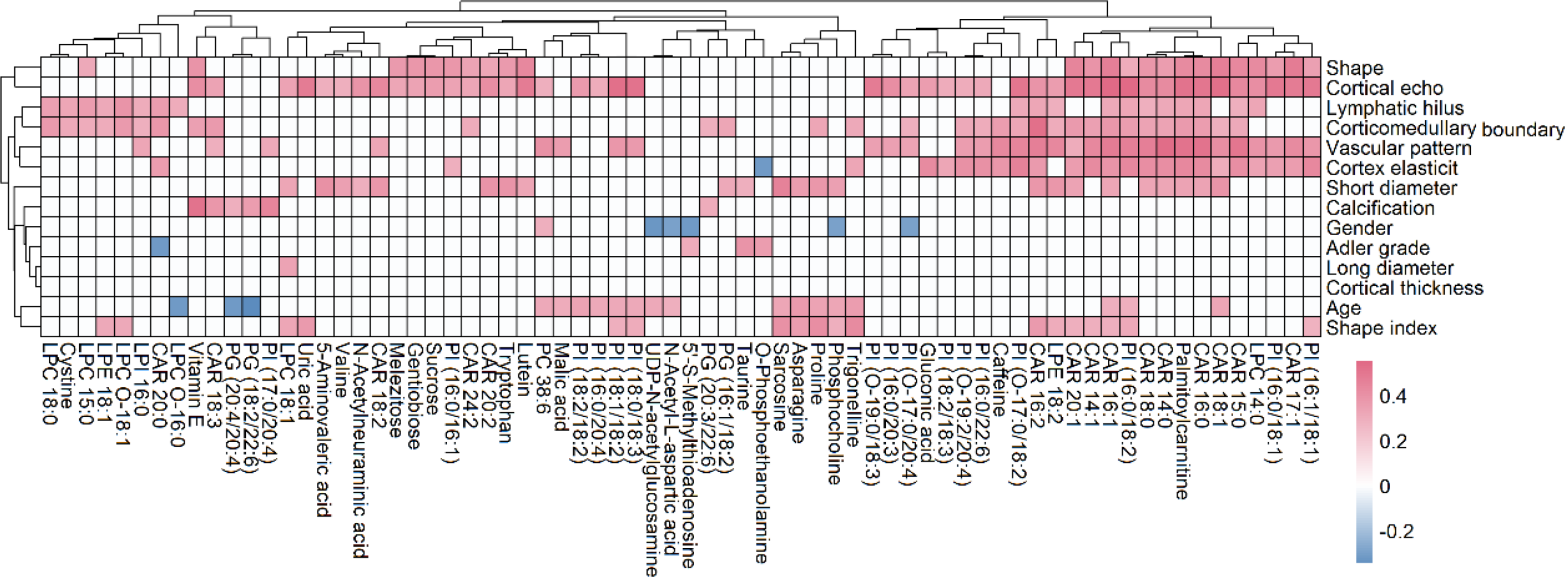
Correlation analysis of metabolites with ultrasound features and patient indices (age and gender), where the metabolites with more than 2 significant correlations were shown. The red and blue blocks represent significant positive and negative correlations (*P* < 0.05), respectively, and the white color indicates metabolites with no significant correlation (*P* > 0.05).

Pearson’s coefficient was used to determine the correlations of identified metabolites with ultrasound features and patient indices (age and gender). To simplify interpretation, the *P*-value was limited to a low of 0.05. Our results showed that many metabolites were significantly correlated with patient indices and ultrasound features. 366 significant correlations were detected, of which 343 were positive and 23 were negative (**Figure 7**). There existed 25, 26, 27, 27, 36, 21, 47, 37, and 13, correlated with age, short diameter, shape, cortex elasticity, blood flow mode, hilus, cortical echo, corticomedullary boundary and calcification, respectively. Specifically, many CARs, such as CAR 16:0, CAR 16:1 and CAR 18:0, were positively correlated with short diameter, shape, cortex elasticity, blood flow mode, hilum, cortical echo and corticomedullary boundary. Many PIs, such as PI (16:0/18:2), PI (16:0/20:3), PI (16:0/22:6), PI (16:1/18:1) and PI (18:0/18:3), were positively correlated with blood flow mode and cortical echo. Eight PGs, such as PG (18:2/20:4), PG (18:2/20:3) and PG (20:4/22:4), were significantly positively with calcification. For carbohydrates, sucrose, gentiobiose and melezitose were positively correlated with shape and cortical echo, and glucose was positively correlated with corticomedullary boundary. Some amino acids, such as N-acetylneuraminic acid, tryptophan and valine, were positively correlated with cortical echo. Asparagine, trigonelline, proline, phosphocholine, sarcosine, UDP-N-acetylglucosamine were positively correlated with age (r > 0.3, *P* < 0.05).

## 4 Discussion

Conventional ultrasound is considered to be the first choice for evaluating lymph node diseases due to its high resolution (5). Although different lymph node lesions have characteristic signs on ultrasound images, there are some overlapping images of different pathological types of lymph nodes. For example, both inflammatory and lymphatic cancers may be enlarged, spherical, and often irregularly outlined (6). Therefore, the pathological diagnosis of lymph nodes is still a challenging subject in ultrasound diagnosis (15). Our results showed that age, vascular pattern and cortical echo could be applied to distinguish malignant and benign lymph nodes and shape, cortex elasticity and corticomedullary boundary was important variance to distinguish metastasis and lymphoma groups. Our finding was in line with the result of Cui et al. (5). An echopoor in the lymph node cortex may indicate lymphoma, whereas a hyper echo in cortical may indicate metastasis lymph nodes. Peripheral or mixed vascularity mode suggested malignant lymph nodes, however, lymphoma lymph nodes could remain mixed vascularity or high flow hilar vascularity.

Metabolomics reveals differences in the metabolic profiles of the three groups of lymph node tissue (**Figure 2A**), which may indicate that cancer cells could change their metabolism for rapid proliferation and expansion. Amino acids are not only the building blocks of proteins, but also play a regulatory role in key metabolic cascades, gene expression and intercellular communication in human’s cells (16). Our results showed that compared to the benign group, the metastasis and lymphoma groups are characterized by higher levels of many amino acids, such as glutamine, asparagine, histidine, lysine, methionine, devaline, threonine, and valine. It has been reported that many amino acids can be absorbed into tumor tissues from non-cancer cells adjacent through cell surface transporters (17, 18). These increase amino acids could support cell growth and metabolism of malignant lymph nodes, not only aiding in protein biosynthesis, but also helping to maintain redox balance as an energy source in addition to glucose (16). For example, in malignant lymph nodes, glutamine could drive the citrate cycle to maintain mitochondrial ATP production and act as a carbon and nitrogen donor for purine biosynthesis (19). Methionine can affect the epigenetic state of cancer cells and promote the development of tumors (20). There is growing evidence that amino acid restriction can inhibit cancer cell growth and may improve the efficacy of chemotherapeutic agents (21). Glutamic acid and pyroglutamic acid increased significantly in the metastasis group, about twice as much as in the benign and lymphoma groups. Glutamic acid could be used as bioenergy substrate for cell growth (22, 23) and pyroglutamic acid, involved in the metabolism of glutathione, could produce glutamic acid via 5-oxproline enzyme (24). This finding suggested that the lymphoma group is more dependent on glutamate metabolism than the metastasis group. Other three amino acids, N-acetyl-L-aspartic acid, N-acetylneuraminic acid and tryptophan, were additionally accumulated in metastasis groups, compared to the other two groups, most of which have a prominent role in cancer growth and participates in the immunosuppressive effects (25, 26). The three amino acids could act potential biomarkers for diagnosis and therapy of metastasis superficial lymph nodes. Histamine, a biogenic amine synthesized from histidine, was significantly accumulated in metastasis groups, about 3 times as much as in the benign and lymphoma groups (27). This suggested that histamine might be involved in metastasis carcinogenesis. Our finding was in line with precious results that some human cancers such as ovarian, cervical, and endometrial cancers have high histamine levels compared to adjacent normal tissues (19). It has been reported that most malignant cell lines express their own histamine-synthesizing enzyme to enhance the level of endogenous histamine, which could be released into the space between cells (27). The above data indicated that histamine may be a crucial mediator in the development and progression of metastasis superficial lymph nodes and should be a good target for the cancer therapy.

Organic acids play key roles in many intracellular metabolic pathways, such as amino acid synthesis and metabolism, glycolytic metabolism, and cholesterol biosynthesis (28). Four organic acids, including malic acid, citric acid, pipecolic acid and P-aminobenzoic acid, were increased in both lymphoma and metastasis groups, compared to benign groups. Among them, malic acid and citric acid are intermediate metabolites in the citrate cycle, which can produce cell energy and precursors of biosynthetic pathways (29). Pipecolic acid is a minor metabolite of lysine catabolism (30). These findings suggest that both lymphoma and metastasis superficial lymph nodes lead to abnormal metabolism of lysine and citrate cycle, which may be a common biomarker for malignant lymph node therapy. Besides, uric acid, 5-aminovaleric acid and benzoic acid were additionally accumulated in metastasis groups, all of which have been reported as biomarker in cancer cells (31–34). In particular, uric acid is a degradation product of purines that was common chemical compound found in foods and drinks (35). When tumor cells occurred DNA-damaged, tumor cells also lead uric acid accumulation, due to its antioxidant function. However, as the uric acid levels increased, the risk of cancer might be raised (36, 37). Methylmalonic acid, a metabolic intermediate in the biosynthesis of succinic acid from propionic acid (38), were additionally decreased in lymphoma groups compared to the other groups, and could act as a potential biomarker for diagnosis lymphoma.

Carbohydrates are the main source of energy in most human diets (39). Our results showed that glucose levels in both the metastasis and lymphoma groups were significantly higher than those in the benign group, suggesting abnormal glucose metabolism in the malignant lymph nodes (40). The finding is consistent with previous reports that cancer cells need more glucose than normal cells (41). In general, cancer cells usually survive in low-oxygen environments because they overgrow and become dense (42). However, cancer cells are less efficient at breaking down sugars in a hypoxic state than in aerobic conditions, making them require more glucose to produce energy for survival and proliferation (43). Besides, other four carbohydrates, sucrose, gentiobiose, raffinose, and melezitose, were additionally accumulated in metastasis groups, compared to the other two groups, which could act as a potential biomarker for diagnosis of metastasis lymph nodes. Our finding was partly supported by precious studies that sucrose can be used to distinguish papillary thyroid cancer and multinodular goiter (44). Correlation analysis revealed that four carbohydrates may have effect on the shape of lymph nodes. Recent studies have proven that higher carbohydrate intake not only speeds up the absorption of carbohydrates by cancer cells, but also stimulates rapid cancer cell reproduction (39). These data suggest that the occurrence of metastasis cancer may be related to excessive carbohydrate intake.

Lipids are not only important components of cell membranes, but also participate in many biological functions such as energy storage and signaling (44, 45). Our results showed that 95 lipids showed significant changes among different kinds of lymph nodes, mainly including fatty acids, lysophospholipids, glycerophospholipids and acylcarnitines. Many lipids increased in metastasis and lymphoma groups compared to benign groups, which may be due to the increased uptake or synthesis of lipids in the cancer cell to meet the demand of the high nutrient and energy needs during cancer growth progression (46, 47). This suggested that both lymphoma and metastasis superficial lymph nodes had abnormal lipid metabolism. Besides, our result revealed that the metastasis group was characterized by higher levels of CAR, PI and PG. CAR facilitates the transportation of fatty acids into the mitochondria and higher levels of CAR may indicate an elevated level of lipid oversupply in the metastasis group (48). High levels of PG may indicate mitochondrial dysfunction and enhanced invasion ability of metastasis cells (49). Additionally, some metabolite related to glycerophospholipid metabolism was found, among which phosphocholine, CDP-choline, acetyl-L-Carnitine and carnitine were increased in both lymphoma and metastasis groups, compared to benign groups. Taken together, our results reveal differences of lipid synthesis and metabolism in different kinds of malignant lymph nodes and these lipids and their associated metabolites with significant change could be used as potential diagnostic and therapeutic targets. Correlation analysis also revealed that the levels of 8 PIs were significantly correlated with the calcification of lymph nodes. This finding was in line with precious studies that PI3K/AKT pathways phosphorylating PI is closely related to vascular calcification, osteogenesis and osteoclast formation (50). Besides, many CAR and PI were positively correlated with shape, blood flow mode, cortical echo and corticomedullary boundary and these relationships require further proof.

Nucleotide metabolism plays an important role in cancer progression (51). Our results showed that many nucleosides were increased in both lymphoma and metastasis groups compared to benign groups, including two purines (hypoxanthine and guanine) and four nucleotides (adenosine 3’-monophosphate, UDP-D-glucose, UDP-N-acetylglucosamine and flavin adenine dinucleotide). Most of them could be beneficial for rapid cancer cell proliferation (52–54). For example, both hypoxanthine and guanine can replenish the purine pool of proliferative cancer cells (55). UDP-N-acetylglucosamine is produced through the hexosamine biosynthesis pathway, which promotes rapid tumor growth (56). The increase of this metabolite may indicate activation of the hexosamine-biosynthetic pathway in malignant tissues (57). Besides, other three nucleotides, guanosine monophosphate, adenosine diphosphate and inosine monophosphate, were additionally accumulated in metastasis groups, compared to the other groups and inosine monophosphate has the greatest increase at more 3-folds. Inosine monophosphate plays a central role in purine metabolism in cells and acts as a precursor to the synthesis of adenosine monophosphate and guanosine monophosphate, suggesting that inosine monophosphate could act as potential biomarkers for diagnosis and therapy of lymphoma lymph nodes (58). Additionally, two metabolites (guanosine and uracil) were additionally increased at 2-folds in metastasis groups, compared to the other two groups. Guanosine shows the effect of inducing differentiation in cancer cells and uracil is one of the four nucleotide bases in RNA (59). The above data suggested that metastasis lymph nodes have effect of the pathway of guanosine and uracil metabolism.

Linear discriminant analysis showed that the correct diagnosis rate of metabolomics based on random forest for variable selection was much better than that of ultrasonography (63.1%~75.4%). Compared to ultrasound indices, metabolites were more accurate in determining the type of malignant lymph nodes. The finding suggests that metabolomics coupled with linear discriminant analysis could serve as an effective approach for lymph node diagnosis. Furthermore, metabolomics coupled with deep learning models for feature extraction and classification warrants further investigation. However, it is important to note that metabolomics requires tissue sampling, which indicates that the metabolomic approach cannot be used for real-time assessment of lymph nodes. In contrast, ultrasonography is a non-invasive technique, which could effectively monitor the status of lymph nodes in real time. The quantitative analysis of characteristic metabolites in lymph nodes could be developed based on non-destructive methods, such as spectroscopy and ultrasonography. Furthermore, incorporating key metabolites into the ultrasonography model may enhance the diagnostic accuracy in disease detection.

## 5 Conclusion

In the present study, we evaluated the differences in benign, lymphoma and metastasis superficial lymph nodes using ultrasonography and metabolomics. Via metabolomics, we observed significant increases in many amino acids, organic acids, lipids, and nucleosides in both the lymphoma and metastasis groups, compared to the benign group. Specifically, the lymphoma group exhibited higher levels of nucleotides (inosine monophosphate, guanosine monophosphate, and adenosine diphosphate) as well as glutamic acid and the metastasis group was characterized by higher levels of carbohydrates, acylcarnitines, glycerophospholipids, uric acid, and 5-aminovaleric acid. The correct diagnosis rate of these metabolites for differentiating superficial lymph nodes ranged from 87.4% to 89.3%, which is more effective than ultrasound indicators. This study is limited by its small sample size and the absence of an external validation set. Future research will focus on increasing the sample size and validating the clinical significance of the identified potential biomarkers. Additionally, we will investigate the metabolic changes occurring in lymph nodes throughout disease progression.

## Data Availability

The original contributions presented in the study are included in the article/**Supplementary Material**. Further inquiries can be directed to the corresponding authors.
