# Supplementary material for: Discrimination of superficial lymph nodes using ultrasonography and tissue metabolomics coupled with machine learning
Source: Front Oncol. 2025 Jan 28;15:1510018. doi: 10.3389/fonc.2025.1510018 (PMC11810734; doi:10.3389/fonc.2025.1510018)
Supplement: Supplementary file 1 [file DataSheet1.docx]

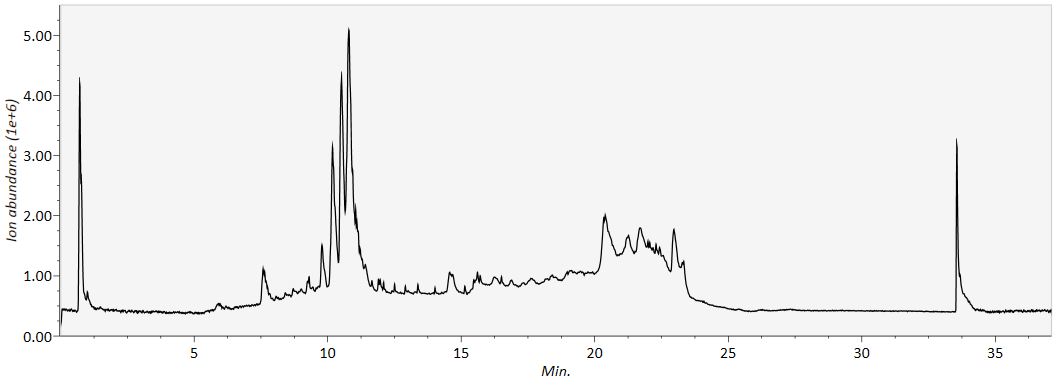


Fig. S1 LC-QTOF/MS chromatogram graph of a lymph node sample in the positive mode.


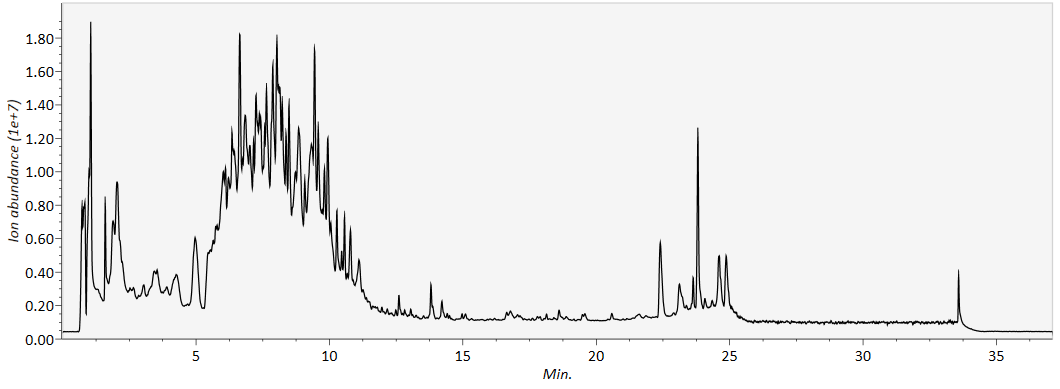


Fig. S2 LC-QTOF/MS chromatogram graph of a lymph node sample in the negative mode.

Table S1 Information about the MS peaks identified.

| Time | Average Mz | Adduct type | Metabolites | Class |  | Area intensity |  |
| --- | --- | --- | --- | --- | --- | --- | --- |
|  |  |  |  |  | Benign | Lymphoma | Metastasis |
| 0.702 | 191.0613 | [M-H]- | Quinic acid* | Organic acids | 1.3E+03 | 2.4E+03 | 2.4E+03 |
| 0.703 | 191.016 | [M-H]- | Citric acid* | Organic acids | 1.3E+03 | 2.4E+03 | 2.4E+03 |
| 0.722 | 184.9846 | [M-H]- | 2-Phosphoglycerate | Lipids | 2.2E+02 | 1.5E+02 | 5.3E+02 |
| 0.728 | 126.1018 | [M+H]+ | 1-Methylhistamine | Alkaloids | 2.3E+02 | 4.1E+02 | 5.3E+02 |
| 0.735 | 112.0874 | [M+H]+ | Histamine* | Alkaloids | 5.0E+02 | 5.1E+02 | 1.4E+03 |
| 0.743 | 133.0176 | [M-H]- | Malic acid* | Organic acids | 9.4E+02 | 1.3E+03 | 2.0E+03 |
| 0.789 | 132.0272 | [M-H]- | Aspartic acid* | Animo acids | 2.4E+03 | 4.3E+03 | 4.2E+03 |
| 0.796 | 565.0425 | [M-H]- | Uridine 5'-diphospho-D-glucose | Nucleosides | 2.8E+03 | 8.3E+03 | 5.6E+03 |
| 0.807 | 195.0515 | [M-H]- | Gluconic acid* | Organic acids | 2.4E+03 | 1.5E+03 | 3.1E+03 |
| 0.811 | 137.0334 | [M-H]- | p-Hydroxybenzoic acid | Organic acids | 2.5E+02 | 1.8E+02 | 1.9E+02 |
| 0.816 | 606.0675 | [M-H]- | Uridine-5-diphosphoacetylglucosamine | Nucleosides | 6.4E+03 | 1.9E+04 | 2.2E+04 |
| 0.818 | 175.1177 | [M+H]+ | Arginine* | Animo acids | 5.7E+03 | 8.2E+03 | 1.1E+04 |
| 0.827 | 308.0927 | [M-H]- | N-Acetylneuraminic acid | Animo acids | 6.7E+02 | 9.0E+02 | 1.8E+03 |
| 0.843 | 179.0582 | [M-H]- | Glucose* | Sugars | 1.5E+03 | 2.4E+03 | 3.5E+03 |
| 0.844 | 133.06 | [M+H]+ | Asparagine* | Animo acids | 9.8E+02 | 2.0E+03 | 2.2E+03 |
| 0.845 | 179.0551 | [M-H]- | Inositol | Other | 1.7E+03 | 2.4E+03 | 3.3E+03 |
| 0.849 | 124.008 | [M-H]- | Taurine | Amino acids | 7.3E+03 | 1.2E+04 | 1.1E+04 |
| 0.853 | 241.0298 | [M+H]+ | Cystine* | Animo acids | 1.1E+03 | 2.4E+03 | 2.6E+03 |
| 0.855 | 184.0729 | [M+H]+ | Phosphocholine* | Alkaloids | 1.0E+04 | 3.4E+04 | 4.4E+04 |
| 0.862 | 90.05511 | [M+H]+ | Sarcosine | Animo acids | 8.9E+02 | 2.0E+03 | 2.4E+03 |
| 0.863 | 147.0754 | [M+H]+ | Glutamine* | Animo acids | 5.2E+03 | 9.8E+03 | 1.0E+04 |
| 0.863 | 147.1074 | [M+H]+ | Lysine* | Animo acids | 5.1E+03 | 9.7E+03 | 1.0E+04 |
| 0.866 | 117.0175 | [M-H]- | Methylmalonic acid | Organic acids | 1.1E+03 | 3.7E+02 | 4.8E+02 |
| 0.868 | 142.0264 | [M+H]+ | O-Phosphoethanolamine | Alkaloids | 3.5E+03 | 7.7E+03 | 4.9E+03 |
| 0.883 | 118.0519 | [M-H]- | Threonine* | Animo acids | 3.1E+02 | 4.8E+02 | 5.4E+02 |
| 0.883 | 130.0972 | [M+H]+ | Pipecolic acid* | Organic acids | 7.5E+03 | 1.5E+04 | 1.1E+04 |
| 0.884 | 130.0504 | [M+H]+ | Pyroglutamic acid | Animo acids | 8.9E+03 | 1.7E+04 | 1.4E+04 |
| 0.893 | 148.0603 | [M+H]+ | Glutamic acid* | Animo acids | 3.0E+04 | 5.9E+04 | 4.2E+04 |
| 0.906 | 162.1133 | [M+H]+ | Carnitine | Alkaloids | 1.6E+04 | 2.6E+04 | 3.2E+04 |
| 0.954 | 489.1126 | [M+H]+ | Cytidine 5'-diphosphocholine | Alkaloids | 1.4E+03 | 7.3E+03 | 5.6E+03 |
| 0.96 | 154.0594 | [M-H]- | Histidine* | Animo acids | 8.9E+02 | 1.5E+03 | 1.8E+03 |
| 0.971 | 360.1515 | [M+NH4]+ | Gentiobiose | Sugars | 2.0E+02 | 2.5E+02 | 5.6E+02 |
| 0.979 | 138.0545 | [M+H]+ | Trigonelline | Alkaloids | 4.5E+02 | 5.0E+02 | 6.5E+02 |
| 0.981 | 116.0719 | [M+H]+ | Proline* | Animo acids | 1.3E+04 | 2.4E+04 | 3.9E+04 |
| 0.998 | 426.0173 | [M-H]- | ADP | Nucleosides | 4.4E+02 | 1.1E+03 | 4.2E+02 |
| 0.999 | 203.1482 | [M+H]+ | N,N-Dimethylarginine | Animo acids | 4.0E+03 | 7.5E+03 | 7.2E+03 |
| 0.999 | 362.0503 | [M-H]- | Guanosine 5'-monophosphate | Nucleosides | 6.8E+02 | 2.3E+03 | 1.2E+03 |
| 1.007 | 527.1567 | [M+Na]+ | Melezitose | Sugars | 1.8E+03 | 1.5E+03 | 6.0E+03 |
| 1.008 | 365.105 | [M+Na]+ | Sucrose* | Sugars | 1.3E+03 | 1.5E+03 | 3.1E+03 |
| 1.008 | 503.1538 | [M-H]- | Raffinose | Sugars | 1.0E+02 | 1.0E+02 | 2.8E+02 |
| 1.011 | 347.0375 | [M-H]- | Inosine-5-monophosphate | Nucleosides | 8.7E+02 | 5.7E+03 | 2.1E+03 |
| 1.026 | 116.0724 | [M-H]- | 5-Aminovaleric acid | Organic acids | 1.0E+03 | 1.4E+03 | 2.5E+03 |
| 1.089 | 118.0867 | [M+H]+ | Valine* | Animo acids | 3.5E+03 | 5.6E+03 | 6.6E+03 |
| 1.252 | 348.0674 | [M+H]+ | Adenosine 3'-monophosphate | Nucleosides | 3.9E+03 | 2.0E+04 | 9.8E+03 |
| 1.256 | 204.1212 | [M+H]+ | Acetyl-L-Carnitine | Alkaloids | 1.5E+04 | 2.8E+04 | 3.3E+04 |
| 1.256 | 152.0575 | [M+H]+ | Guanine* | Nucleosides | 6.7E+03 | 1.3E+04 | 1.2E+04 |
| 1.263 | 176.054 | [M+H]+ | N-Acetyl-L-aspartic acid | Animo acids | 4.8E+02 | 7.9E+02 | 1.6E+03 |
| 1.269 | 138.0501 | [M+H]+ | P-Aminobenzoic acid | Organic acids | 3.1E+03 | 4.9E+03 | 5.0E+03 |
| 1.274 | 169.0347 | [M+H]+ | Uric acid* | Organic acids | 4.6E+03 | 5.8E+03 | 7.8E+03 |
| 1.275 | 180.0619 | [M-H]- | Tyrosine* | Animo acids | 4.8E+02 | 7.9E+02 | 9.4E+02 |
| 1.277 | 150.0576 | [M+H]+ | Methionine* | Animo acids | 3.5E+03 | 5.6E+03 | 7.4E+03 |
| 1.288 | 307.9773 | [M+H]+ | Glutathione* | Animo acids | 5.3E+01 | 8.4E+01 | 8.6E+01 |
| 1.294 | 123.0553 | [M+H]+ | Niacinamide | Alkaloids | 3.9E+03 | 7.2E+03 | 7.0E+03 |
| 1.357 | 151.0259 | [M-H]- | Xanthine | Nucleosides | 2.1E+02 | 3.1E+02 | 3.9E+02 |
| 1.37 | 137.047 | [M+H]+ | Hypoxanthine | Nucleosides | 3.9E+04 | 6.2E+04 | 6.1E+04 |
| 1.428 | 118.0813 | [M+H]+ | Norvaline | Animo acids | 8.4E+02 | 1.0E+03 | 9.7E+02 |
| 1.512 | 136.0567 | [M+H]+ | Adenine* | Nucleosides | 7.9E+01 | 9.3E+01 | 9.0E+01 |
| 2.061 | 664.119 | [M+H]+ | beta-Nicotinamide adenine dinucleotide | Nucleosides | 1.0E+01 | 2.5E+01 | 3.1E+01 |
| 2.11 | 113.0308 | [M+H]+ | Uracil | Nucleosides | 2.3E+02 | 3.2E+02 | 4.7E+02 |
| 2.56 | 123.0383 | [M+H]+ | Benzoic acid | Organic acids | 1.2E+02 | 1.5E+02 | 2.0E+02 |
| 2.871 | 138.0909 | [M+H]+ | Tyramine* | Alkaloids | 9.0E+01 | 9.8E+01 | 9.9E+01 |
| 3.414 | 269.0877 | [M+H]+ | Inosine | Nucleosides | 2.9E+02 | 4.3E+02 | 5.6E+02 |
| 3.606 | 268.1007 | [M+H]+ | Adenosine* | Nucleosides | 1.9E+02 | 1.4E+02 | 2.2E+02 |
| 4.796 | 282.0823 | [M-H]- | Guanosine* | Nucleosides | 3.8E+01 | 4.7E+01 | 8.2E+01 |
| 5.397 | 209.0865 | [M+H]+ | Kynurenine | Organic acids | 4.1E+02 | 6.4E+02 | 6.7E+02 |
| 5.674 | 165.9926 | [M+H]+ | Phenylalanine* | Animo acids | 2.6E+01 | 2.9E+01 | 3.3E+01 |
| 7.117 | 205.0978 | [M+H]+ | Tryptophan* | Animo acids | 9.2E+02 | 1.4E+03 | 3.5E+03 |
| 7.169 | 298.097 | [M+H]+ | 5'-S-Methylthioadenosine | Nucleosides | 1.6E+03 | 4.8E+03 | 4.6E+03 |
| 7.487 | 439.1009 | [M+H]+ | Flavin Adenine Dinucleotide | Nucleosides | 2.8E+02 | 4.4E+02 | 5.5E+02 |
| 8.012 | 195.0882 | [M+H]+ | Caffeine | Other | 1.7E+02 | 1.6E+02 | 3.0E+02 |
| 8.462 | 121.0298 | [M-H]- | 4-Hydroxybenzaldehyde | Other | 4.1E+02 | 3.2E+02 | 3.8E+02 |
| 8.624 | 681.2341 | [M-H]- | Pinoresinol diglucoside | Other | 1.6E+01 | 2.2E+01 | 1.8E+01 |
| 9.999 | 749.5149 | [M+H]+ | Azithromycin | Other | 7.3E+00 | 2.8E+03 | 1.0E+01 |
| 12.2 | 157.1244 | [M-H]- | FA 9:0 | Lipids | 6.4E+02 | 6.4E+02 | 9.1E+02 |
| 15.763 | 595.296 | [M-H]- | LPI 18:2 | Lipids | 1.4E+02 | 2.2E+02 | 2.2E+02 |
| 15.86 | 370.2934 | [M+H]+ | CAR 14:1 | Lipids | 2.5E+02 | 3.0E+02 | 1.2E+03 |
| 15.868 | 619.2847 | [M-H]- | LPI 20:4 | Lipids | 7.5E+02 | 1.6E+03 | 1.2E+03 |
| 16.101 | 318.2983 | [M+H]+ | Phytosphingosine | Lipids | 1.2E+03 | 1.3E+03 | 3.7E+03 |
| 16.435 | 571.2841 | [M-H]- | LPI 16:0 | Lipids | 4.1E+02 | 3.9E+02 | 1.1E+03 |
| 16.454 | 396.3075 | [M+H]+ | CAR 16:2 | Lipids | 4.8E+02 | 4.6E+02 | 1.1E+03 |
| 16.505 | 199.1671 | [M-H]- | Lauric acid | Lipids | 2.2E+03 | 1.9E+03 | 2.6E+03 |
| 16.587 | 621.304 | [M-H]- | LPI 20:3 | Lipids | 1.7E+02 | 2.8E+02 | 4.1E+02 |
| 16.855 | 372.3114 | [M+H]+ | CAR 14:0 | Lipids | 2.3E+03 | 2.3E+03 | 1.4E+04 |
| 16.91 | 468.3103 | [M+H]+ | LPC 14:0 | Lipids | 4.6E+02 | 5.0E+02 | 2.1E+03 |
| 17.107 | 422.3263 | [M+H]+ | CAR 18:3 | Lipids | 4.3E+02 | 3.7E+02 | 8.8E+02 |
| 17.227 | 509.2873 | [M-H]- | LPG 18:1 | Lipids | 4.3E+02 | 9.9E+02 | 1.0E+03 |
| 17.278 | 398.3253 | [M+H]+ | CAR 16:1 | Lipids | 1.8E+03 | 1.7E+03 | 8.7E+03 |
| 17.482 | 494.3227 | [M+H]+ | LPC 16:1 | Lipids | 8.1E+02 | 7.7E+02 | 2.6E+03 |
| 17.507 | 386.3254 | [M+H]+ | CAR 15:0 | Lipids | 3.1E+02 | 2.5E+02 | 1.1E+03 |
| 17.571 | 559.3001 | [M-H]- | LPG 22:4 | Lipids | 1.6E+02 | 2.6E+02 | 1.9E+02 |
| 17.65 | 478.2911 | [M+H]+ | LPE 18:2 | Lipids | 2.9E+03 | 2.8E+03 | 5.6E+03 |
| 17.71 | 424.3417 | [M+H]+ | CAR 18:2 | Lipids | 7.5E+03 | 5.1E+03 | 1.4E+04 |
| 17.748 | 502.2928 | [M+H]+ | LPE 20:4 | Lipids | 2.8E+04 | 3.8E+04 | 3.9E+04 |
| 17.851 | 482.3225 | [M+H]+ | LPC 15:0 | Lipids | 3.9E+02 | 3.1E+02 | 6.3E+02 |
| 17.883 | 412.3431 | [M+H]+ | CAR 17:1 | Lipids | 1.3E+02 | 1.1E+02 | 4.4E+02 |
| 18.182 | 528.3013 | [M+H]+ | LPE 22:5 | Lipids | 4.6E+03 | 6.9E+03 | 6.5E+03 |
| 18.189 | 400.3424 | [M+H]+ | CAR 16:0 | Lipids | 2.4E+04 | 1.7E+04 | 7.6E+04 |
| 18.235 | 400.3399 | [M+H]+ | Palmitoylcarnitine | Lipids | 2.4E+04 | 1.7E+04 | 7.3E+04 |
| 18.364 | 504.3098 | [M+H]+ | LPE 20:3 | Lipids | 1.0E+03 | 1.5E+03 | 2.1E+03 |
| 18.525 | 426.3589 | [M+H]+ | CAR 18:1 | Lipids | 1.2E+04 | 9.6E+03 | 5.0E+04 |
| 18.63 | 273.2212 | [M-H2O+H]+ | Epiandrosterone | Other | 1.0E+02 | 1.2E+02 | 1.3E+02 |
| 18.632 | 320.1382 | [M+H]+ | Norfloxacin | Other | 4.1E+01 | 3.4E+01 | 4.7E+01 |
| 18.774 | 480.3117 | [M+H]+ | LPE 18:1 | Lipids | 3.2E+03 | 3.8E+03 | 5.3E+03 |
| 18.91 | 452.3775 | [M+H]+ | CAR 20:2 | Lipids | 9.1E+02 | 8.8E+02 | 3.7E+03 |
| 19.036 | 522.3532 | [M+H]+ | LPC 18:1 | Lipids | 5.6E+03 | 7.6E+03 | 1.2E+04 |
| 19.119 | 530.3252 | [M+H]+ | LPE 22:4 | Lipids | 5.1E+03 | 7.0E+03 | 6.6E+03 |
| 19.498 | 428.373 | [M+H]+ | CAR 18:0 | Lipids | 1.1E+04 | 4.8E+03 | 4.0E+04 |
| 19.526 | 482.3604 | [M+H]+ | LPC O-16:0 | Lipids | 1.6E+04 | 5.2E+03 | 1.4E+04 |
| 19.533 | 510.3596 | [M+H]+ | LPC 17:0 | Lipids | 4.1E+02 | 2.8E+02 | 5.4E+02 |
| 19.54 | 772.5695 | [M+H]+ | PC 35:2 | Lipids | 1.3E+03 | 1.0E+03 | 1.0E+03 |
| 19.751 | 454.3889 | [M+H]+ | CAR 20:1 | Lipids | 1.2E+03 | 7.7E+02 | 4.7E+03 |
| 20.886 | 456.411 | [M+H]+ | CAR 20:0 | Lipids | 2.8E+03 | 6.8E+02 | 3.0E+03 |
| 20.998 | 656.4133 | [M-H]- | PE (12:0/18:3) | Lipids | 7.1E+01 | 6.7E+01 | 9.2E+01 |
| 21.084 | 524.3684 | [M+H]+ | LPC 18:0 | Lipids | 4.2E+04 | 2.6E+04 | 4.8E+04 |
| 21.293 | 508.4357 | [M+H]+ | CAR 24:2 | Lipids | 2.2E+02 | 1.3E+02 | 4.0E+02 |
| 21.899 | 508.3732 | [M+H]+ | LPC O-18:1 | Lipids | 9.5E+02 | 3.9E+02 | 8.2E+02 |
| 21.91 | 758.574 | [M+H]+ | PC (16:0/18:2) | Lipids | 6.6E+04 | 6.2E+04 | 5.0E+04 |
| 22.216 | 510.3872 | [M+H]+ | LPC O-18:0 | Lipids | 2.1E+03 | 5.9E+02 | 9.3E+02 |
| 22.539 | 281.252 | [M-H]- | Vaccenic acid | Lipids | 2.2E+02 | 3.8E+02 | 3.6E+02 |
| 22.721 | 855.4981 | [M-H]- | PI (18:2/18:3) | Lipids | 7.3E+01 | 9.3E+01 | 1.4E+02 |
| 22.922 | 732.5535 | [M+H]+ | PC (16:0/16:1) | Lipids | 1.5E+04 | 1.2E+04 | 3.0E+04 |
| 23.042 | 841.5025 | [M-H]- | PG (20:4/22:6) | Lipids | 1.7E+03 | 8.6E+02 | 1.8E+03 |
| 23.119 | 772.6036 | [M+H]+ | PC O-36:2 | Lipids | 3.7E+03 | 2.8E+03 | 2.9E+03 |
| 23.129 | 817.4993 | [M-H]- | PG (18:2/22:6) | Lipids | 3.5E+03 | 1.9E+03 | 3.3E+03 |
| 23.161 | 881.5179 | [M-H]- | PI (18:2/20:4) | Lipids | 1.3E+03 | 1.8E+03 | 2.4E+03 |
| 23.167 | 817.5137 | [M-H]- | PG (20:4/20:4) | Lipids | 3.9E+03 | 2.4E+03 | 4.8E+03 |
| 23.21 | 831.5048 | [M-H]- | PI (16:0/18:3) | Lipids | 7.3E+02 | 4.9E+02 | 1.1E+03 |
| 23.267 | 793.4942 | [M-H]- | PG (18:2/20:4) | Lipids | 4.4E+03 | 2.8E+03 | 4.6E+03 |
| 23.276 | 857.5085 | [M-H]- | PI (18:2/18:2) | Lipids | 1.4E+03 | 2.8E+03 | 4.8E+03 |
| 23.289 | 716.5264 | [M+H]+ | PE (16:0/18:2) | Lipids | 3.6E+03 | 1.3E+03 | 1.2E+03 |
| 23.324 | 867.5142 | [M-H]- | PG (22:5/22:6) | Lipids | 3.0E+03 | 2.0E+03 | 3.5E+03 |
| 23.359 | 806.5581 | [M+H]+ | PC 38:6 | Lipids | 4.2E+03 | 6.0E+03 | 6.4E+03 |
| 23.38 | 843.5146 | [M-H]- | PG (20:3/22:6) | Lipids | 3.6E+03 | 2.6E+03 | 4.9E+03 |
| 23.399 | 781.4904 | [M-H]- | PI (14:0/16:0) | Lipids | 3.6E+02 | 3.5E+02 | 9.4E+02 |
| 23.418 | 843.5187 | [M-H]- | PG (20:4/22:5) | Lipids | 3.9E+03 | 2.6E+03 | 4.9E+03 |
| 23.422 | 769.5045 | [M-H]- | PG (18:2/18:2) | Lipids | 6.2E+03 | 5.4E+03 | 9.1E+03 |
| 23.441 | 881.5372 | [M-H]- | PI (O-19:2/20:4) | Lipids | 2.3E+03 | 2.5E+03 | 7.6E+03 |
| 23.441 | 881.551 | [M-H]- | PI (16:0/22:6) | Lipids | 2.2E+03 | 2.4E+03 | 7.1E+03 |
| 23.454 | 857.5171 | [M-H]- | PI (16:0/20:4) | Lipids | 3.9E+03 | 6.6E+03 | 1.3E+04 |
| 23.575 | 807.5064 | [M-H]- | PI (16:0/16:1) | Lipids | 7.0E+02 | 8.4E+02 | 1.0E+04 |
| 23.577 | 819.5118 | [M-H]- | PG (18:2/22:5) | Lipids | 1.1E+04 | 9.0E+03 | 1.4E+04 |
| 23.58 | 819.5138 | [M-H]- | PI (15:0/18:2) | Lipids | 1.0E+04 | 8.9E+03 | 1.4E+04 |
| 23.635 | 857.5566 | [M-H]- | PI (O-17:0/20:4) | Lipids | 3.5E+04 | 3.8E+04 | 8.5E+04 |
| 23.651 | 833.5201 | [M-H]- | PI (16:1/18:1) | Lipids | 3.5E+03 | 5.5E+03 | 1.8E+04 |
| 23.656 | 743.48 | [M-H]- | PG (16:1/18:2) | Lipids | 1.2E+03 | 1.3E+03 | 1.8E+03 |
| 23.705 | 795.5194 | [M-H]- | PG (18:2/20:3) | Lipids | 8.9E+03 | 7.2E+03 | 1.3E+04 |
| 23.748 | 845.5496 | [M-H]- | PG (20:4/22:4) | Lipids | 3.8E+03 | 3.2E+03 | 4.8E+03 |
| 23.753 | 745.5004 | [M-H]- | PG (16:1/18:1) | Lipids | 3.0E+03 | 3.1E+03 | 7.4E+03 |
| 23.757 | 833.5664 | [M-H]- | PI (O-17:0/18:2) | Lipids | 5.9E+03 | 5.9E+03 | 2.1E+04 |
| 23.766 | 745.4993 | [M-H]- | PG (16:0/18:2) | Lipids | 3.0E+03 | 3.1E+03 | 7.4E+03 |
| 23.767 | 833.5134 | [M-H]- | PI (16:0/18:2) | Lipids | 9.0E+03 | 1.0E+04 | 3.1E+04 |
| 23.818 | 795.5174 | [M-H]- | PG (18:1/20:4) | Lipids | 1.0E+04 | 7.4E+03 | 1.4E+04 |
| 23.862 | 771.5189 | [M-H]- | PG (18:1/18:2) | Lipids | 1.9E+04 | 1.7E+04 | 3.3E+04 |
| 23.863 | 859.5046 | [M-H]- | PI (16:0/20:3) | Lipids | 6.8E+03 | 9.8E+03 | 1.7E+04 |
| 23.874 | 909.5479 | [M-H]- | PI (20:2/20:4) | Lipids | 5.6E+03 | 9.4E+03 | 7.4E+03 |
| 23.882 | 859.5592 | [M-H]- | PI (18:1/18:2) | Lipids | 1.4E+04 | 2.1E+04 | 3.7E+04 |
| 23.888 | 859.5684 | [M-H]- | PI (O-19:0/18:3) | Lipids | 1.3E+04 | 2.0E+04 | 3.4E+04 |
| 23.894 | 859.5353 | [M-H]- | PI (18:0/18:3) | Lipids | 1.4E+04 | 2.2E+04 | 3.8E+04 |
| 23.933 | 804.557 | [M+Na]+ | PC 36:4 | Lipids | 7.9E+02 | 1.2E+03 | 1.0E+03 |
| 24.002 | 797.5382 | [M-H]- | PG (18:1/20:3) | Lipids | 5.0E+03 | 5.6E+03 | 1.2E+04 |
| 24.036 | 693.5035 | [M-H]- | PG (14:0/16:0) | Lipids | 3.5E+02 | 4.2E+02 | 1.6E+03 |
| 24.077 | 871.5309 | [M-H]- | PI (17:0/20:4) | Lipids | 5.8E+03 | 5.2E+03 | 7.3E+03 |
| 24.111 | 799.5517 | [M-H]- | PG (18:0/20:3) | Lipids | 2.0E+03 | 1.3E+03 | 4.3E+03 |
| 24.225 | 747.5179 | [M-H]- | PG (16:0/18:1) | Lipids | 6.9E+03 | 1.1E+04 | 4.6E+04 |
| 24.234 | 719.4832 | [M-H]- | PG (16:0/16:1) | Lipids | 1.3E+03 | 1.6E+03 | 8.3E+03 |
| 24.36 | 885.5712 | [M-H]- | PI (18:0/20:4) | Lipids | 3.7E+04 | 6.7E+04 | 6.6E+04 |
| 24.371 | 773.5313 | [M-H]- | PG (18:1/18:1) | Lipids | 1.1E+04 | 1.4E+04 | 2.7E+04 |
| 24.426 | 835.5563 | [M-H]- | PI (16:0/18:1) | Lipids | 1.1E+03 | 1.7E+03 | 3.7E+03 |
| 24.439 | 799.5415 | [M-H]- | PG (18:1/20:2) | Lipids | 1.4E+03 | 1.8E+03 | 4.2E+03 |
| 25.907 | 568.428 | [M]+ | Lutein | Vitamins | 3.5E+01 | 3.9E+01 | 6.5E+01 |
| 30.552 | 429.3726 | [M-H]- | Vitamin E | Vitamins | 2.1E+02 | 1.7E+02 | 3.1E+02 |

*, confirmed by the standard.
